# Observations and Remarks upon Softening of the Brain

**Published:** 1841-04

**Authors:** 


### Art. X.

*Beobachtungen und Bemerkungen über Gehirnerweichung.* Von Dr. C. H. Fuchs, Professor der Medicin zu Würzburg.—*Leipzig*, 1838. 8vo, pp. 245.

*Observations and Remarks upon Softening of the Brain.* By Dr. C. H. Fuchs, Professor of Medicine at Wurzburg.—*Leipsic*, 1838.

Encephalomalacia, ramollissement, or softening of the brain, notwithstanding the researches of Rostan, Lallemand, Herbst, Gendrin, Abercrombie, and Carswell, seems scarcely to have met with that attention which the frequency of its occurrence, in connexion with the paralysis of old people, and the extreme fatality attending it, require. The work of M. Rostan was the first in which it was made known to the profession that there is a peculiar disorganization of the cerebral structure, consisting in a greater or less degree of softening or breaking down of some portion of the brain, which, in many instances at least, is capable of being recognized during the life of the patient by the symptoms accompanying it. M. Rostan's cases occurred in persons of advanced age, and present many features in common with other cerebral affections to which those in the decline of life are liable; but Dr. Abercrombie showed that the pathological change occurs more frequently, according to his experience, in the young, and especially in connexion with acute hydrocephalus, either as a complication or as a specific form of that disease. The author of the work before us also describes cases of softening of the brain occurring in young persons as well as in the aged, and from his observations it would seem that, while the cases of the latter description resemble more nearly those related by M. Rostan, those of the former are for the most part of a somewhat different character, and to be considered rather as secondary affections consequent upon the development of some other diseased process.

The treatise before us embraces nine chapters, severally devoted to the following subjects: 1, The Pathological Anatomy; 2, Cases of idiopathic or primary Softening, and its complications with sanguineous and serous effusion; 3, the Symptoms; 4, the Diagnosis, and cases of secondary softening; 5, the Causes; 6, the Duration, Progress, and Terminations; 7, the Prognosis; 8, the Therapeutics; and 9, the Nature of the disease.

In the first chapter, under the head of pathological anatomy, a pretty full account is given of the morbid change which constitutes the essential characteristic of this peculiar affection. This change consists in a breaking down of the structure of some portion of the brain, cerebellum, or spinal cord, by which the cerebral or cerebro-spinal matter loses its natural filamentous appearance, and becomes more or less discoloured, softened, and infiltrated, in some instances being reduced to a completely disorganized semi-fluid pulp. The alteration of colour is described by Dr. Fuchs as varying from milk-white, dull-white, or opalescent, or very pale and dirty yellow, to greenish or brownish yellow, and not unfrequently to reddish, deep, dusky, or brown red, brown, and even approaching to black; of consistence from that in which the usual appearance is presented to the eye and the degeneration of structure is recognized only by the readiness with which the cerebral matter is reduced to pulp by the slightest impression of the finger, to that in which it is found already in a pultaceous or, as has been observed by MM. Andral and Velpeau, an altogether fluid condition. The variations of consistence would seem to indicate either the intensity or the duration of the morbid process, while those of colour are probably derived, in part at least, from more or less injection or infiltration of the softened part with blood, lymph, serum, or other fluids. The whitish and yellow-coloured softening of the brain, is thought by M. Lallemand, who considers this morbid alteration of structure as a product of inflammation, to be owing to purulent infiltration in the cerebral texture, and the red or brown coloured softening as deriving its tints from inflammatory congestion. Dr. Fuchs, however, while he admits that the change of colour in the latter class of instances is owing to the presence of blood in the altered and disorganized structure, does not consider that the congestion is necessarily inflammatory in its origin.

"Every instance of softening of the brain," he observes, "must, according to the views of Lallemand, Gendrin, and others, be red coloured at its commencement, that is, inflammatory; and the pale, faded, yellow softening, which is synonymous with suppuration, should occur only as a consequence of the former state. But to me the colourless, pale, or faded softening clearly seems the most simple condition; while, on the contrary, the reddish, red, wine-lees discoloration is formed, when parts of the brain abounding in blood are affected, or where the softening, from whatever cause, coexists with congestion, through destruction of the vessels in the softened part, and more or less infiltration of the disorganized matter with extravasated blood; finally, the dark discoloration is a consequence of the sanguineous softening." (p. 5.)

The extent of the morbid change is liable to great variation. Dr. Fuchs states that he has seen instances in which the softening did not exceed the size of a cherry-stone, while in others almost the whole of one of the cerebral hemispheres has been involved in the disease. Other writers speak of the entire brain as having been softened, but it has not fallen within the experience of the author of this treatise to observe a case of idiopathic encephalomalacia of such extent. The softening spreads itself sometimes superficially, sometimes deeply in the substance of the brain, and sometimes equally in every direction. Occasionally it is diffused throughout the different cerebral structures; in other instances it is found either in the white or cineritious portions, or confined to a single convolution, or to the corpus striatum or thalamus nervorum opticorum of one side. M. Rostan states that the corpora striata and optic thalami are the parts which are most frequently the seat of the softening; but if the cases in which this diseased condition is complicated with other diseases of the brain be excluded, it will be found that some part of one or other of the hemispheres, chiefly the right, is most usually affected. Of the first thirty cases mentioned by M. Rostan, which are those of the simple uncomplicated disease, both hemispheres were affected in four; in thirteen the right hemisphere alone was the chief seat of the morbid alteration, and the left in seven. The cases narrated by Dr. Fuchs afford a similar result; the right side of the brain being the seat of the destructive process in nine cases, the left side suffering only in four.

A frequent morbid change of structure found by M. Rostan, and one which is regarded by Dr. Carswell as a well-ascertained and frequent cause of the disease as it occurs in advanced life, is obstruction of the circulation in the brain from the deposition of osseous or fibrous matter in the arteries at the base. Dr. Fuchs, however, has had occasion to observe this structural derangement, in this situation, in two only of his cases; but in two others there was ossification of the arch of the aorta; in a fifth case there was deposition of bony matter in the valvular apparatus of the left side of the heart as well as in the arch of the aorta; and in a sixth the abdominal portion of this vessel was ossified.

The cases related in the second chapter are fourteen in number, of which six are examples of the simple and uncomplicated disease, the softened portions of the brain being but slightly tinged with blood, and serous effusion, if present, in inconsiderable quantity. In two cases the infiltration of the disorganized cerebral structure with blood was considerable, and was attended with a corresponding variation in the symptoms; in two others there was sanguineous effusion into the softened part producing genuine apoplexy; and in another the disease was complicated with copious effusion of serum. The three remaining cases are related as instances of a favorable termination of the disease; the subject of one of these, however, subsequently died from an attack of pneumonia, and on examination the softening of the brain was distinctly recognized, so that, although the symptoms were so far relieved as to allow of the dismissal of the patient from the hospital, the disease can scarcely be said to have been cured. In the other two no verification of the diagnosis having taken place some doubt must remain as to their being genuine instances of the affection.

Dr. Fuchs, as others have done before him, recognizes three stages of the disease: 1st, a precursory stage, in which the symptoms are upon the whole much the same as those which are found to precede many other forms of cerebral disease; 2d, the stage of paralysis; and 3d, a stage of low or torpid fever which follows with greater or less rapidity the occurrence of the paralytic symptoms. These several stages are, however, not always to be distinctly made out, temporary attacks of paralysis of slight and partial character sometimes arising in the precursory stage, and gradually passing into a more extensive or more permanent affection of this character before the loss of power or sensation becomes complete; while, in other instances, the typhoid state very rapidly follows upon that of paralysis. In Dr. Fuchs's cases the first stage was altogether wanting in one; and in three others it could not be distinctly ascertained whether the attack of paralysis had been preceded by premonitory symptoms or not; in the remaining cases the duration of this period was found to vary much, the extremes being twenty-four hours and six or seven months. In some of M. Rostan's cases the precursory stage would seem to have been of much longer duration.

Pain of head, usually but not always accompanied with confusion and giddiness; pale and sunken appearance of the countenance; dull and muddy eye, without heat of scalp, and a small and weak pulse, are enumerated among the most frequent symptoms of this stage, although they are by no means always present. Dr. Fuchs remarks that in almost all the cases in which the pain of head occurred, there was, in addition to the softened state of the brain, either extravasation of blood or effusion of serous fluid. In two cases only was the headach fixed, continuous, and severe, in both of which chronic rheumatism (arthritis) had preceded the softening, and examination after death proved the existence of cephalic rheumatism "in the thickened membranes, ossified arteries, and small exostoses, the products of this affection in the head." Disturbance of the intellectual faculties was not common in this stage, and a disordered condition of one or more of the senses only observed as accompanying the headach. The most constant precursory symptom was some irregularity, diseased sensation, or deficient power in the organs of voluntary motion, in other words, incipient paralysis. "Almost every patient," says Dr. Fuchs, "experienced, sooner or later before the commencement of the second stage, if only occasionally, a sense of weight, weakness, numbness, and aching in the extremities of one side, that which subsequently became paralysed, and usually more marked in the leg than in the arm." Contraction of the muscles of one of the limbs, or of the extremities of one half of the body, was not observed by Dr. Fuchs among the premonitory symptoms, but convulsive motions of the muscles of the face and extremities, with strong tremor, were noticed in one case several months before the appearance of the fatal symptoms, and slighter and more partial attacks of the same nature occurred in two other instances. A diagnostic symptom to which the author attaches considerable importance was observed in three of the cases; it consists in a sudden loss of power in the extremities of one side while walking, so that the patient is compelled to sit down, or falls, without suffering any loss of consciousness. The circulation is but little affected in this stage, and the functions of nutrition and assimilation scarcely if at all disturbed.

The second stage of the disease is ushered in by an attack of decided paralysis, the paralytic symptoms being continuous and usually confined to one side, although in one of Dr. Fuchs's cases in which the softening was ultimately found to be seated in the *corpus callosum*, it extended to both sides of the body. Of the cases related in the work before us, in nine the paralysis was on the left side, and in four on the right, corresponding to the situation of the softening on the opposite sides of the brain. The attack was usually sudden, and in one case only did the paralytic symptoms come on so gradually as to render the line of demarcation between the first and second stages difficult to be distinguished. In two of the cases the attack occurred in the middle of the night, in nine in the early part of the day, and in two only, in which the softening was complicated with effusion of blood, in the afternoon.

The paralysis is not always complete at the outset, and hence the author has been led to recognize two varieties of the disease, a more acute and a more chronic form, and he thinks that the complication with sanguineous or serous effusion will usually be found to belong to the more acute variety. The first attack of paralysis also does not in all cases continue without change until the fatal termination of the disease, an evident amelioration being occasionally observed, and sometimes a complete restoration of the paralysed limbs. Loss of sensation as well as of motion, as in other forms of paralysis, is occasionally observed. Contraction of the muscles of the paralysed limb, a symptom considered by M. Rostan as pathognomic, was noticed by Dr. Fuchs in three cases only; in these the paralysed limbs were bent, the flexor muscles feeling firm and contracted. In each case this symptom commenced at the same time with the paralysis; in one of the cases the muscles did not become relaxed until death, in a second the contraction together with the paralytic symptoms ceased a short time before the fatal termination, and in the third, one of those the result of which was favorable, the relaxation of the muscles took place a few days after the first traces of returning power were observed. The paralytic affection is not confined to the limbs, but extends also to the face and other parts of the body, in the majority of instances the mouth being drawn to one side, the cheek hanging, the eyelid dropped, the speech stammering and inarticulate. In one case there was slight difficulty of swallowing, and in three others complete dysphagia. Usually when the paralysis of the extremities was or had been complete the urine passed involuntarily, and the bowels were obstinately constipated. The consciousness, on the contrary, was seldom entirely extinguished at the outset of the paralysis, and, as it would appear, only in those cases in which there was complication with extravasation of blood; in some of the cases, on the contrary, the intellects seemed at first but little impaired for some time; and in one instance the loss of consciousness was not complete until about the fourteenth day from the attack of paralysis. The speech was frequently affected, but those who could make themselves understood complained of pain of head, especially on the side opposite the paralysed limbs; the pain was described by one patient as lancinating, shooting through the head, and intermitting; by others as pressing or stabbing. In only one case there was no pain of head. All who were able to speak complained of giddiness and great confusion of the head, and many of muscæ volitantes and sparks before the eyes and of noises in the ears. In every case of uncomplicated softening the face was pale, the expression of countenance troubled, the head did not feel hotter than the rest of the body, the eyes were dull, muddy, and hollow, neither vascular nor suffused, and the pupils of the natural size. The respiration was free and without rattle, although, in the more intense cases, quickened; the pulse was somewhat more frequent, never slower than natural, small, feeble, occasionally unequal, and in one case weaker in the paralysed arm than in the other. The temperature of the skin was usually unchanged, the tongue for the most part clean and moist, the thirst moderate, the appetite not much impaired, and the secretions and excretions both in quality and quantity commonly but little disordered. In the cases complicated with extravasation of blood, there was, on the contrary, more or less evidence of a state of congestion,—such as heat of scalp, pulsation of the arteries, and swelling of the veins of the head, lividity of the lips and tongue, stertorous respiration, &c.; and, when there was effusion of serum, loss of consciousness, puffiness of the face, dilated pupils, with impeded respiration, and rapid, small, irregular pulse.

The duration of this stage varied in Dr. Fuchs's cases from a few hours to twenty-five days, but the distinction between the second or paralytic and the third or febrile stage does not appear to be always clearly marked. Febrile symptoms indeed sometimes occurred in the premonitory stage, and occasionally death supervened in a few hours from the first onset of the paralytic state. In two of the three successful cases also the febrile symptoms never appeared at all, the one recovering in nine days, the other in a few months. The third stage is characterized by febrile irritation; small, weak, irregular pulse, with burning heat and dryness of skin; darkly furred, dry tongue; delirium and stupor, &c. This state commonly terminates fatally in from two to twelve days.

Dr. Fuchs sums up the characteristics of the simple idiopathic softening of the brain as follows:

1. *Premonitory stage.* A sense of debility over the whole body, of heaviness, numbness, and loss of power in the extremities, usually of one side, mental disquiet, and muddy pallid complexion are its most constant symptoms. The morbid sensations in the extremities are sometimes more lasting, the patients having a constant sensation as if the limbs were asleep; they drag more in walking, and are unable to use the arm and hand with as much freedom and strength as usual; but more frequently these symptoms are only occasionally present, coming on rather in paroxysms, as it were, in which the extremities of one side suddenly fail, and the patient must either sit down or fall but in a few seconds or minutes is again able to rise and pursue his way. In addition to these symptoms the patient complains occasionally of headach, giddiness, stammering, sparks before the eyes, noise in the ears, &c. and the powers of the mind are impaired, though all these last symptoms are often absent. The circulation and vegetative functions are undisturbed during this stage.

2. *Paralytic stage.* This stage usually comes on suddenly and for the most part early in the morning, the precursory symptoms but seldom passing by degrees and without a marked paroxysm into the state of paralysis. The patient generally becomes hemiplegic, and falls down with his mouth drawn and deprived of the use of the limbs of one side, more frequently of the left than of the right. In many cases the paralysis of the limbs is complete immediately on the first attack; in others, on the contrary, there is at first more or less limited motion, without strength or power of support, and the loss of power passes gradually with the further progress of the disease into perfect paralysis. Occasionally the paralysed limbs are at the same time without sensation, sometimes the sense of feeling continues strong; in many cases they are the seat of severe lancinating pains, and in others the flexor muscles are tense, hard, and contracted by tonic spasm or cramp, but seldom do they become affected in clonic or convulsive spasm. The consciousness is occasionally lost immediately at the onset, but in the greater number of cases it becomes extinct later towards the end of the second or during the progress of the third stage. All the patients, however, complain of confusion, giddiness, wandering, and many point to the head, especially to the side opposite to the paralysed one, as in pain. All are more or less confused and deaf, comprehending the questions addressed to them more slowly than in health, stammering and faltering in their speech, and often inarticulate and not to be understood; in several the speech fails altogether. The power of swallowing is occasionally lost, and from paralysis of the organs of the pelvis, the stools are for the most part retained, and the urine passed involuntarily. All appearances of congestion and determination of blood are absent; the face is pale and collapsed, the temperature of the head and of the whole body normal, the eye dull without suffusion or vascularity and hollow, the pupils unchanged; the respiration is easy and tranquil, and the pulse small, feeble, sometimes of the usual frequency, often somewhat accelerated, unequal, and irregular.

3. *Febrile stage.* The fever sets in with dry, burning-hot skin, small irregular pulse, darkly-coated dry tongue, dirty incrustation of the teeth, fuliginose discharge from the nostrils, and with great prostration of strength. If the consciousness had before been but little disturbed, wandering delirium takes place, which soon terminates in sopor: if the confusion had been previously considerable, the patient immediately becomes comatose. He is speechless and no longer capable of being roused; the paralysis is complete, and all perception of external impressions lost. Finally, the respiration becomes accelerated, laborious, and occasionally noisy, and death, generally in a few days, terminates the scene. (p. 115.)

In the fourth chapter the diagnosis between softening of the brain and various allied diseases, more especially apoplexy with sanguineous and serous effusion, inflammatory affections of the brain and its membranes, and certain chronic cerebral diseases, tubercle, cancer, &c. is pointed out. Dr. Fuchs draws an admirable parallel between the symptoms of softening on the one hand, and those of sanguineous apoplexy on the other; and were the softening always uncomplicated with congestive symptoms, as in the summary above given, little difficulty would be experienced; but here, as in almost every department of practical medicine, disease seldom presents itself at the bedside with the same simplicity as in the descriptions of authors. Much nice discrimination is therefore necessary; close examination of the symptoms, and careful weighing of their respective indications, before the complications of disease can be accurately unravelled. Still there are certain leading features by which the thoughts of the observant practitioner may be directed into the right channel; thus, with respect to the discrimination between obscure cases of primary softening of the brain complicated with extravasation of blood, and cases of sanguineous apoplexy in which softening of the cerebral structure becomes a secondary morbid degeneration consequent upon the extravasation of blood, the circumstances of age, time of attack, and order of symptoms, will afford good grounds whereupon to found a conclusion, although the general expression of the disease may be obscured.

The form of softening to which our attention has hitherto been exclusively directed occurs, for the most part, in persons of very advanced life, and chiefly among females of irritable or debilitated constitution. Of M. Rostan's first thirty cases, which are those of simple uncomplicated ramollissement, the ages of seven were between sixty and seventy; of fifteen between seventy and eighty; and of eight, eighty and upwards; three only were below the age of sixty-seven. Of Dr. Fuchs's cases, there were four under seventy and ten above that age. The attack of paralysis marking the commencement of the second stage usually sets in in the earlier part of the day; and, as was pointed out by M. Rostan, coma, which is the first symptom of apoplexy, is commonly the last in encephalomalacia. In apoplexy, in addition to the deep coma and stertorous respiration which characterizes the attack, those who are the subjects of it are usually males of less advanced age and more robust habit, and the time of seizure is commonly in the afternoon or at night after a full meal.

The distinctive marks between softening and other forms of apoplexy are then pointed out. The greater number of recorded cases of the so-called nervous apoplexy are considered by Dr. Fuchs, in accordance with the opinion expressed by M. Rostan, to have been owing to softening of some portion of the brain. Serous apoplexy, which he terms hydrocephalus senilis, may readily be recognized, he thinks, by the absence of the peculiar premonitory symptoms before described, of the contraction of the muscles, pain of the limbs, &c. Of far greater difficulty and importance is it to distinguish between this affection, when it occurs in children or young persons, and the hydrocephalus of early life, if indeed they are not rather to be considered as different terminations of the same disease. It is certain that softening occurring in the young is preceded by or accompanied with symptoms far more nearly allied to those of inflammation of the brain in its membranes than those usually observed in the same affection of the aged; and those who have attentively studied Dr. Abercrombie's cases and compared them with the results of their own experience, cannot fail to perceive that the earlier symptoms of acute hydrocephalus and encephalomalacia in the infant or young adult are absolutely the same; in short, both the effusion of serum on the one hand, and the softening on the other, are, in these cases, merely consequences of a previous inflammatory state, and not unfrequently found combined: they are both secondary affections arising out of a previous disease.

Dr. Fuchs remarks upon this subject, that in children and young persons who die of acute hydrocephalus, the walls of the distended lateral ventricles, the septum lucidum, the fornix, &c. are, in the greater number of cases, reduced to a milk-white disorganized (faserlose) pulp; and states that he never saw this morbid change absent when the stages of congestion and effusion were followed by a third stage, in which the hitherto-slow pulse became hurried and not to be counted, the skin burning hot, the tongue dry and darkly coated, and the last traces of consciousness were lost. This state is commonly characterized by convulsions ushering in paralysis, sometimes confined to one side of the body, but usually general, to which death for the most part in a short time succeeds. On the other hand, two cases are mentioned in which death took place in the second stage, the pulse being still slow and the skin cool, where upon inspection a considerable collection of water was found but no softening of the brain.

We have already expressed our opinion as to the difficulty of distinguishing the complications of softening from other affections of the cerebral organs. Dr. Fuchs, however, is sanguine enough to conclude that not only may primary or idiopathic encephalomalacia be with certainty detected by the character and succession of the symptoms, but that it may with tolerable accuracy be predicted where secondary softening has taken place in other affections of the brain. He proceeds to point out at some length the symptoms and marks by which, as he conceives, the existence of this peculiar morbid degeneration may be conjectured, but as these are confessedly derived from a very limited experience, and are not altogether in accordance with what has been observed by others, it is unnecessary here to refer to them further.

The fifth chapter is devoted to the consideration of the causes which, in the author's opinion, exert an influence in producing or favouring the disease. Among these he enumerates advanced age, debility of constitution, however induced, irritability of temperament, a state of poverty and wretchedness, mental emotions, the abuse of spirituous liquors, extremes of temperature, &c. A diseased state of the vessels of the brain, as we have before remarked, does not appear to him of the same importance in relation to the disease as it occurs in those of advanced age, as to some other observers ; and with respect to the secondary disease, although its frequent occurrence in connexion with the hydrocephalus of children and the inflammatory conditions of the brain in young or middle-aged adults is admitted, the author does not consider it as otherwise connected with inflammation than as a consequence of the debilitating influence of that affection, or of the treatment necessarily had recourse to in its progress. To him, softening of the brain, whether idiopathic or secondary, appears to be induced, " by debilitating, paralysing influences, namely, by such as circumscribe the vegetative life of the brain," (p. 189;) and he concludes the treatise by declaring that with Hopfengaertner he considers " encephalomalacia as a peculiar asthenic injury of the brain with annihilation of the local organic life (vegetation)." (p. 245.)

If there is any difference between the sentiments here expressed and those of other observers who recognize the occasionally inflammatory nature of this affection, beyond that of mere words, we are ourselves completely at issue with Dr. Fuchs ; but if he merely means to say that the disorganization of structure where it arises in connexion with a previous inflammatory state, results from the destructive effects of such a state upon the powers and organization of the affected part, no one, we believe, will be inclined to contest his position. The fact appears to be, that softening of the brain, as explained by Dr. Abercrombie and Dr. Carswell, is a form of gangrene, or rather sphacelus, which may arise in this organ as elsewhere from various causes acting injuriously on the local vitality and organization. The two principal of these causes are inflammation of the cerebral substance, and obstruction of the circulation from obliteration of the cerebral vessels. In the latter class of cases we hold the non-inflammatory character of the affection to be completely established. It is, in fact, a form of gangrena senilis. The following case, related by Dr. Abercrombie and occurring in a young person, is an exceedingly instructive one, as the symptoms were chiefly those of simple idiopathic softening, as described by MM. Rostan and Fuchs; and the peculiar diseased condition of the basilar artery affords a very sufficient explanation of the occurrence of the softening at so early an age without marks of inflammation:

"A young man, aged eighteen, had been for six or eight weeks affected with cough and pain of chest, and was supposed to be phthisical; but for several days he had been much better, when on the 15th December, 1819, he suddenly fell down deprived of sense and motion, and paralytic on the left side, with twisting of the mouth. When partially recovered, he complained of severe pain in the right temple; his speech was very indistinct; countenance expressive of great stupor. The usual treatment was actively employed, but without much benefit, and he continued for about ten days with little or no improvement; the left side perfectly paralytic; a great degree of coma; the speech very indistinct; but he still pointed to the right temple as the seat of fixed uneasiness. During this time his pectoral complaints had completely disappeared. In January, 1820, he began to improve, so as to have less uneasiness in his head and considerable motion of the leg; but the arm continued entirely paralytic. His cough now returned, with considerable pain in the right side of the chest. He continued without further change till the 15th of February, when he complained of pain in the back of his head, and was seized with loss of speech and of the power of swallowing. He soon recovered his speech; but the power of swallowing was permanently lost, so that from this time he was constantly fed by liquids introduced into the stomach through an elastic gum tube. He was now quite distinct, and did not complain of any pain; the cough again abated; pulse of natural frequency but feeble. In the beginning of March he seemed to improve a little in strength, so that he was several times taken out in a carriage: there was considerable motion of the left leg, but the arm continued perfectly paralytic; no return of the power of swallowing; speech and intellect entire. He died rather suddenly on the 20th of March, having the day before become extremely weak and pale without any obvious cause. *Inspection.* On removing the dura mater, there appeared on the middle of the right hemisphere a remarkable depression, which, when cut into, was found to arise from an extensive mass of pure ramollissement; the part being in the state of a soft white pulp, without any appearance of pus and without fetor; it extended the whole depth of the hemisphere. In the cerebral matter adjoining to this disease, there was a small abscess no larger than a bean, lined with a firm soft cyst of coagulable lymph. There was very little effusion in the ventricles, and no other disease in the substance of the brain. On raising the brain a remarkable appearance was found in the basilar artery; through the extent of about an inch it was very much enlarged and hard, and this portion was found to be completely filled up by a firm white matter without any appearance of blood. Anterior to this portion there was a small coagulum of blood in the artery. The lungs were tolerably healthy; but there was a considerable deposition of coagulable lymph, forming a thick firm mass betwixt the right lung and the pleura costalis at the lower part immediately above the diaphragm."

This case, as it seems to us, affords an example of this peculiar degeneration arising from obstructed circulation, and is analogous, therefore, to the same affection as it occurs in old people; the greater number of Dr. Abercrombie's cases, together with some few of those related by M. Rostan, the majority of M. Lallemand's, and those described by Dr. Fuchs as secondary encephalomalacia, are, on the contrary, of inflammatory origin, and present an alteration of structure analogous to the destructive effects produced by inflammation in other parts where the vitality is destroyed.

The observations in the sixth chapter on the duration, progress, and terminations of the disease, are anticipated by what has already been said; and with respect to the prognosis and treatment, which form the subject of the seventh and eighth chapters, it is unnecessary to say more than that the former is for the most part in the highest degree unfavorable, and the latter hitherto inefficacious. The nature of the disease we have taken occasion to point out in the course of the preceding observations; and it only remains for us to express our approbation of the manner in which Dr. Fuchs has treated his subject.

